# Serum Levels of Matrix Metalloproteinase-1 in Brazilian Patients with Benign Prostatic Hyperplasia or Prostate Cancer

**DOI:** 10.1155/2020/6012102

**Published:** 2020-05-05

**Authors:** William Khalil El-Chaer, Audrey Cecília Tonet-Furioso, Gilberto Santos Morais Junior, Vinícius Carolino Souza, Gleiciane Gontijo Avelar, Adriane Dallanora Henriques, Clayton Franco Moraes, Otávio Toledo Nóbrega

**Affiliations:** ^1^Universidade de Brasília, Brasília, DF, Brazil; ^2^Universidade Católica de Brasília, Águas Claras, DF, Brazil

## Abstract

Metalloproteinases (MMPs) are involved in metastatic tumor processes, with changes in circulating levels detected in several cancer types. Here, we compare serum concentrations of metalloproteinase-1 (MMP-1) across individuals clinically diagnosed with prostate cancer (PCa) or benign prostatic hyperplasia (BPH), correcting results for the rs495366 single nucleotide polymorphism (SNP) that predisposes to differential MMP-1 levels. 196 men aged ≥50 years were followed at a university hospital urology outpatient clinic, with clinical, anthropometric, and rectal examinations performed by one urologist. Blood samples obtained prior to any clinical intervention provided baseline MMP-1 and total/free PSA levels as well as metabolic, hormonal, and inflammatory markers. The SNP was genotyped by real-time PCR. Participants with medical and/or laboratory profile compatible with malignancy composed the PCa group when confirmed by the Gleason scale. As expected, A-allele homozygotes showed reduced levels of MMP-1. Genotype-adjusted analyses revealed the mean MMP-1 level as 2-fold higher in PCa carriers compared to BPH patients. No other differences were found according to the prostatic condition or genotypic distribution, except for the expected raise in total and free PSA levels in PCa. In conclusion, increased serum levels of MMP-1 were observed in this context of prostatic malignancy compared to a benign phenotype, regardless of a genetic influence.

## 1. Introduction

Prostate cancer (PCa) is the second most prevalent type of cancer (31.7%) and has the second highest mortality rate (13.5%) in men in Brazil, with approximately 75% of cases occurring after the age of 65. The National Cancer Institute in Brazil (INCA) estimated 68,220 new cases and 14,484 deaths from PCa in 2018, with the growing incidence observed in recent years being justified by greater access of patients to diagnostic testing and improved notification policies and practices in the health system, along with increasing life expectancy [1].

Early diagnosis of PCa remains critical, as therapeutic resources and the possibility of cure are limited at advanced stages. Medical societies around the world argue that screening efforts promote a shared decision between the physician and the patient, although public policies from several countries (including Brazil) do not support such screening. Clinical management establishes that the presence of visible hematuria, erectile dysfunction, and/or changes in the urinary pattern may warrant investigation for early diagnosis [2, 3].

Currently, PCa screening is usually initiated by the measurement of the prostate-specific antigen (PSA) in conjunction with rectal examination, composing a procedure with sensitivity roughly at 80%. Despite being a low-invasive and inexpensive strategy, it has limited specificity to attest PCa (31% for white patients and 44% for black patients) at the current threshold of 4.0 ng/dL [4]. Serum PSA levels may be elevated in benign prostatic hyperplasia (BPH) or in traumatic and inflammatory prostate conditions, and management based solely on such scores may result in a false-positive result. In fact, only 25% of prostate biopsies motivated by high PSA alone are confirmed as prostate cancer [5].

New markers have been proposed as a second test when PSA titers are altered or borderlined, either alone or in formulas, to increase the specificity of PCa screening. These tests should have a higher predictive value than the use of PSA alone. The free/total PSA ratio and the dosage of PCA3 antigen along with the 4K scores, the prostate health index (PHI), the RC3, and the STOCKHOLM-3 model are alternatives already being used. However, none have yet shown significant gain in clinical accuracy [6, 7].

Studies indicate that the class of serum proteinases of matrix metalloproteinase (MMP) nature is importantly involved in PCa, with potential utility for the entity's diagnosis [8]. MMPs are zinc- and calcium-dependent endopeptidases that degrade various elements of the extracellular matrix (ECM), especially collagen, elastin, laminin, fibronectin, and proteoglycans, and take part in physiological processes involving tissue remodeling. On the other hand, they also contribute to the proliferation and implantation of tumor cells, as well as to angiogenesis [9, 10].

Twenty-four MMPs have already been identified, 23 of them are found in humans, including collagenases (MMP-1, MMP-8, MMP-13, and MMP-18) and gelatinases (MMP-2 and MMP-9). They are found in all tissues and fluids, being usually expressed as membrane-bound pro-MMPs that end up secreted in activated forms by the urokinase-plasminogen/plasmin system located in cell membranes [10].

In particular, the active form of MMP-1 acts by degrading interstitial collagen (including types I, II, and III), with greater expression in the gallbladder and appendix. Located at 11q22.2 where an MMP gene cluster is located, its gene has allelic forms that have been associated with disorders such as lung cancer [11], osteoarthritis [12], and ischemic stroke [13]. Previous studies indicate the possibility of MMP-1 gene promoter polymorphisms associated with PCa [14–16], although a recent meta-analysis concluded that only the MMP-3 11715A/6A and the MMP-9 rs17576 variations were correlated with an increased risk of PCa [17]. Some studies investigated serum levels of MMP-1 in PCa, highlighting the report by Jung et al. [18], who compared different stages of neoplasia (including a BPH group) without producing evidence of differential levels.

Based on experimental evidence that overexpression of MMP-1 in PCa increases neoplastic cell migration and invasion [19], the present study aimed to measure circulating free MMP-1 concentrations of individuals with PCa and BPH, correcting the results for an important allelic variability identified as predisposing to differential serum enzyme levels [20].

## 2. Methods

This is a cross-sectional study developed in male patients consecutively followed at the Outpatient Urology Service of the University Hospital of Brasília, Brazil. Each individual underwent a clinical protocol focused on the characterization of prostatic changes, if any. In addition, clinical, biochemical, metabolic, anthropometric, and inflammatory aspects of each participant were analyzed.

### 2.1. Urological Evaluation

Patients aged 50 years and above underwent an initial urological evaluation according to the criteria of current Brazilian guidelines for the diagnosis of PCa that align with those followed worldwide [21]. The initial clinical procedure consisted in obtaining the patient's clinical history and encompassing signs and symptoms classically associated with active PCa such as dysuria, intermittency of urine stream, straining to urinate, nocturia, sensation of incomplete emptying, and hemospermia. Then, prostate-specific antigen (PSA) levels were obtained for each participant, followed by digital rectal examination (DRE) through which the volume and symmetry of the prostate gland were detected. Patients with altered prostate morphology detected by means of rectal examination and/or elevated PSA levels (>4.0 ng/mL), with or without other signs and symptoms, were referred for biopsy. Biopsies, when indicated, were performed by means of a systematic 12-core ultrasound-guided sampling procedure.

Based on the procedures described, patients with positive biopsy for malignant neoplasia according to the Gleason score (≥6) and that were clinically eligible for watchful surveillance or radical prostatectomy surgery were enrolled in the analyses as cases of PCa [22]. If the suspicion of PCa was excluded based on biopsy, the participant was rendered as a case of benign prostatic hyperplasia as long as an increased prostatic volume was observed with DRE. All cases were assessed by the same physician and were gathered over a period of 36 months.

### 2.2. Laboratory Analyses

Biological samples were collected from peripheral blood of enrolled patients and kept refrigerated at 4°C–8°C for immediate routine biochemical processing or stored frozen at −80°C for further analysis for the present study.

The samples were processed for clinical biochemistry following protocols, quality control, and routine laboratory analytical technical instructions. The glycemic, lipid, enzymatic, metabolic, and inflammatory profiles of each participant were analyzed. Levels of glucose, creatinine, cholesterol, triglycerides, HDL-C, AST (GOT), ALT (GPT), gamma-GT, total proteins, and albumin were measured by enzymatic, kinetic, or colorimetric tests, with reagents compatible with a HumanStar 600 (InVitro®) equipment. The same automation was used to determine ultrasensitive C-reactive protein levels by turbidimetry. Glomerular filtration rate was estimated by the Cockcroft–Gault formula [23], while LDL-C and VLDL-C fractions were estimated by the Friedewald formula [24].

Hematological analyses were performed by automation using ABBOTT® Cell Dyn 3700 equipment, operated according to the manufacturer's recommendations. Glycated hemoglobin was measured by high-performance liquid chromatography (HPLC) technique, while total PSA, free PSA, insulin, vitamin *D*, and TSH levels were obtained by electrochemiluminescence using the Roche® Cobas e411 system. The HOMA beta cell function index and HOMA-IR index were calculated, as well as the free PSA/PSA ratio.

Total circulating MMP-1 was assayed by enzyme-linked immunosorbent assay (ELISA) (R&D Systems—DuoSet, lot 339081), using serum as a sample, and processed according to the manufacturer's guidelines.

### 2.3. MMP-1 Genotyping

Total genomic DNA was obtained using a commercial extraction kit (QIAamp DNA Mini Kit, Qiagen, Brazil). Genotyping reactions were performed using real-time PCR (qPCR) with TaqMan® Universal Master Mix reagent and a specific assay for the rs495366 SNP polymorphism from Thermo Fisher (Massachusetts, USA) based on the stem-loop method. Settings for qPCR started with 50°C for 2 minutes (preread stage) and 95°C for 10 minutes (hold stage) followed by cycling conditions of 95°C for 15 seconds and 60°C for 1 minute for 45 rounds, using the QuantStudio 3 Real-Time PCR System (Thermo Fisher®, Massachusetts, USA).

### 2.4. Statistical Analysis

Student's *t*-test (for parametric data) or the Mann–Whitney test (for nonparametric data) were used to compare the mean (or median) anthropometric and biochemical variables across the carriers of the prostate conditions investigated. For that, close-to-normal distribution of all continuous variables was assessed using the Kolmogorov–Smirnov test. Then, to evaluate the occurrence and strength of the association of circulating levels and of genotypic groups for MMP-1 with classic biomarkers for age-related conditions, correlation coefficients were obtained to unveil the parameters of potential confounding effect in the main model. The association between continuous variables was evaluated using Spearman's correlation test due to the involvement on a regular basis of at least one categorical or nonparametric variable in all analyses. Carriers of different genotypes were represented by consecutive Arabic numbers (for instance, GG, AG, and AA represented by 1, 2, and 3, respectively). Whenever an interaction was noticed, association of the biomarkers with the prostate conditions was tested by means of partial correlation analysis run using adjustment for the confounding variables. Also, raw concentrations of each biomarker were tested across individuals that exhibited HPB or CaP according to our protocol using the Mann–Whitney test. When results showed significant differences, the effect sizes (*d*) and respective confidence intervals (95% CI) were presented. Linear multivariate regression analysis, a stepwise method, was performed to assess to which extent serum concentrations of the biomarkers explain the variability in the occurrence of the prostate condition.

All analyses were performed with the Statistical Package for the Social Sciences (SPSS) for Windows (version 17.0). For this study, the standard two-tailed threshold for significance (*P* ≤ 0.05) was adopted.

## 3. Results

The sample comprised a total of 196 men aged 50 up to 95 years old who provided written consent to participate in the analyses and fulfilled the study protocol by drawing biological samples and providing due clinical data. Of all these individuals, approximately 55% (*n* = 108) were referred for biopsy, whereas the remaining had their clinical condition diagnosed by means of signs and symptoms, prostate morphology, and PSA levels.

Of the cases diagnosed with PCa, findings from rectal examination showed that prostatic alterations were perceptible to touch in most of them (*n* = 19; 95.0%), with changes in consistency (nodules and/or hardening) being the most frequent phenotypes (60% of all PCa cases) and one single case (5.0%) with a non-noticeable change. In marked contrast (*P* < 0.001), similar changes in consistency were only noticeable in a minor proportion (*n* = 12; 6.8%) of patients diagnosed with BPH. At biopsy, the most frequent Gleason score (*n* = 8; 40%) was of the intermediate grade 3 + 4. However, high risk scores (Gleason ≥ 8) represented 30% (*n* = 6) of the total analyzed.

Table 1 presents the mean and median scores of physical and biochemical variables grouped according to prostatic condition. Despite a difference in the number of members, the baseline measurements were very close, indicating clear homogeneity between groups in general clinical conditions. In this context, the equivalent values in terms of C-reactive protein stand out, which precludes possible interference by adverse inflammatory conditions (such as prostatitis, for example) on circulating levels of MMPs [10]. As an expected feature of prostatic neoplasia, a significant difference across groups was observed for total and free PSA levels (as well as in terms of their ratio), regardless that the sensitivity and specificity of PSA for PCa diagnosis are questionable.

Also as expected, the rs495366 genotyping revealed that A homozygotes constituted a minority (6.6%), whereas G homozygotes encompassed nearly half (46.4%) of the patients, with similar mean age and sex ratio across genotypic groups. In the whole sample, genotypic proportions did not deviate from the Hardy–Weinberg equilibrium (*χ*^2^ = 2.6; *P*=0.106) or differed across prostatic condition (*χ*^2^ = 1.9; *P*=0.394).

Based on previous knowledge that this SNP influences serum MMP-1 concentrations [25, 26], the correlations between different genotypic arrangements and levels of the mediator were tested. Also, the correlation of these genotypes/levels with clinical variables (usually deregulated in old age or accentuated in PCa) such as TSH, PSA, and cardiometabolic traits was tested. These tests aimed to evaluate whether serum MMP-1 levels were influenced by allelic variations of the gene or other incidental clinical conditions, with possibility for spurious association with PCa. However, no association was found between SNP or MMP-1 concentration with the clinical traits tested (Table 2). Nevertheless, homozygotes for A-allele showed reduced levels of MMP-1 (*P*=0.045 for rs495366_G_vsAA_), in marked agreement with the best available evidence in the literature.

**Figure 1:**
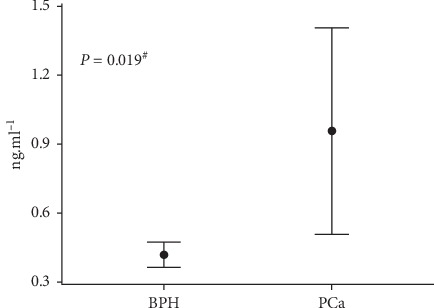
Comparison of raw circulating levels of MMP-1 across individuals diagnosed with benign prostate hyperplasia (BPH) or prostate cancer (PCa). Significance was verified by the partial correlation test controlled for all three rs495366 genotypes. Vertical bars represent intervals of one standard error. ^#^The effect size (*d*) is 1.57, and 95% confidence interval is (0.3; 0.6) for BPH and (0.5; 1.4) for PCa.

Based on the preliminary analyses given above, inferential tests compared MMP-1 levels between patients diagnosed with PCa and with BPH with adjustment for genotypes produced by rs495366 SNP. Thus, despite any influence of genotypes, mean MMP-1 values were significantly different between groups, being exactly 2-fold higher in individuals with PCa (0.96 ng/mL) compared to patients with BPH (0.48 ng/mL) (Figure 1). Analyses following the Cohen convention [27] categorized this difference in MMP-1 levels as of moderate magnitude of effect size (*d*_CaPvsHPB_ = 0.5). Accordingly, a binary logistic regression analysis was performed to ascertain the contributions of both PSA and MMP-1 levels on the likelihood of participants having prostate cancer. The model was statistically significant (*P* < 0.001), explaining 44.8% (Nagelkerke *R*^2^) of the variance in the malignancy and correctly classifying 92.0% of the cases, proportion slightly higher than that from a model using PSA alone (89%). The logistic regression also confirmed that CaP carriers exhibited circulating MMP-1 levels twice as high compared to noncarriers of the malignancy (*P*=0.038).

## 4. Discussion

MMPs compose a family of proteolytic enzymes which are able to degrade cell membrane components and ECM and participate in the expression, production, and secretion of cytokines, adhesion molecules, and cell growth factors. Among the main physiological and pathological processes at stake, MMPs act on embryogenesis, tissue remodeling, angiogenesis, healing, and inflammation [10, 28].

Particularly in cancer, there is an increased expression and release of several MMPs, with consequent increase in tissue and circulating concentrations of these agents and their modulators [29]. Although the biochemical mechanisms by which MMPs take part in tumor progression remain unknown, tumor cells can produce and secrete a diversity of MMPs, an essential fact for the metastatic process [28].

Many studies point to an increase in circulating levels of most MMPs with cancer progression, which makes their measurements promising for use in the diagnosis and prognosis of various types of tumors. Although studies in this regard have been performed, results are conflicting, and it has not been possible so far to define a given MMP with the potential to differentiate any type of cancer [29].

Regarding MMP-1, there are studies in the literature correlating its circulating levels with forms of cancer. Serum MMP-1 levels were measured in lung cancer, being significantly increased in patients with malignancy and positively associated with more advanced stages of the disease and shorter survival [30]. Similar results were achieved in stomach cancer, with elevated circulating levels of MMP-1 and TIMP-1 in patients with this cancer compared to a healthy group, being augmented values associated with aspects of advanced tumor staging such as size, invasiveness, lymph node involvement, liver metastasis, and perineural invasion [31]. In both the cases, the authors suggest a serum dosage of MMP-1 as a prognostic tool and survival predictor factor, regardless of other factors [30, 31].

On the other hand, the mean serum MMP-1 level was found to be decreased in the breast cancer group, in an inverse association with tumor size [32]. Considering the likely role of MMPs in facilitating tumor proliferation and the metastatic process, this apparent contradiction is unexpected and constitutes a fact that is still unexplained in the literature [33].

In PCa, very few studies involving MMP-1 were performed with tissue samples. One of these studies showed direct evidence of MMP-1 involvement in this cancer, with expression in malignant tissues directly proportional to tumor growth rate and metastasis extension, with the use of anti-MMP-1 antibodies inhibiting prostate tumor growth as well as the incidence of pulmonary metastasis in rats [19]. Paradoxically, another study with PCa patients investigated MMP-1 expression in tumoral tissue, with higher *in situ* expression being related to lower Gleason scores, reduced PSA levels, and lower local invasiveness [34].

Regarding MMP-1 as a blood biomarker of PCa, objective of this study, analogous studies are even rarer, which precludes a wider discussion. In one of these studies, plasma MMP-1 levels are compared between healthy men, BPH patients, carriers of PCa *in situ*, and those with metastatic PCa, where mean MMP-1 titers did not differ between groups [18].

Our research aimed to compare serum MMP-1 levels between men diagnosed with PCa and with BPH, in order to test MMP-1's potential as a biomarker for the differential diagnosis of prostate neoplasia. Our results showed that individuals with PCa had the mean MMP-1 levels 2-fold higher. Our analyses minimized the influence of the rs495366 SNP, whose A-allele demonstrated an association with serum MMP-1 levels, validating the finding of significantly elevated MMP-1 values in patients with PCa.

The scarcity of reports on an interrelationship between MMP-1 and PCa precludes proposing that MMP-1 be investigated as a biomarker in PCa. The conflicting results in these few reports make it difficult to advocate for a mechanism of action of this form of metalloproteinase in prostate cancer. Perhaps, at least in part, the conflict stems from the lack of standardization of the technique which is used to quantify MMP, the heterogeneity in the sample, or even the low sampling power of each study. The lack of a rationale to assume a given MMP-1 value as threshold for any clinical entity has made it impossible to calculate sensitivity/specificity or predictive values in our scenario.

Despite the limitations of the study due to its exploratory/preliminary nature and modest recruiting, our results succeeded in associating total circulating MMP-1 levels with prostatic malignancy in a real outpatient setting, particularly prevalent in metabolic disorders [35] and comorbidities [36] (as expected in developing countries) and with the prostate condition of each patient confirmed according to the best possible clinical practice.

## 5. Conclusion

Our study revealed significantly higher serum MMP-1 concentrations in patients with PCa compared to patients with BPH. Further research is needed to corroborate our findings to (if pertinent) allow definition of informative reference or cutoff values for PCa, either for clinical screening or as an accessory confirmatory tool. Clinical follow-up studies with subjects at risk for this malignancy (e.g., family history), aiming to measure circulating MMPs and associate values with the onset and prognosis of prostatic neoplasms, are warranted.

## Figures and Tables

**Table 1:** Clinical data of individuals diagnosed with PCa or BPH.

	PCa (*n* = 20)	BPH (*n* = 176)	*P* ^*∗*^
Age, years	66.0 ± 11.5	67.1 ± 10.2	0.637
BMI, kg/m^2^	26.2 ± 3.8	25.7 ± 4.2	0.624
Glucose, mg/dl	104.3 ± 24.2	102.2 ± 31.7	0.772
HbA1c, %	5.8 ± 1.1	5.8 ± 1.3	0.988
Insulin, mU/mL	7.8 (3.2; 9.2)	5.7 (2.9; 10.0)	0.471^†^
HOMA index	1.8 (0.8; 2.3)	1.4 (0.7; 2.5)	0.440^†^
TGL, mg/dL	141.9 ± 42.1	156.7 ± 113.2	0.249
TC, mg/dL	204.2 ± 37.2	197.4 ± 48.2	0.538
VLDL cholesterol, mg/dL	28.4 ± 8.4	29.2 ± 16.4	0.727
LDL cholesterol, mg/dL	126.5 ± 35.2	114.6 ± 43.7	0.242
HDL cholesterol, mg/dL	49.4 ± 10.8	50.5 ± 13.0	0.710
SGOT, U/L	27.3 ± 11.7	27.6 ± 12.9	0.922
SGPT, U/L	25.6 ± 17.1	26.4 ± 15.4	0.815
GT, U/L	56.0 ± 42.6	49.0 ± 46.3	0.519
Creatinine, mg/dL	1.0 ± 0.2	1.1 ± 0.3	0.585
Total protein, g/dL	7.2 ± 0.5	7.4 ± 0.4	0.192
Albumin, g/dL	4.2 ± 0.4	4.4 ± 0.4	0.216
25-Hydroxy D3, nmol/L	29.4 ± 10.2	31.5 ± 13.1	0.486
CRP, mg/L	1.12 ± 0.77	1.11 ± 0.68	0.995
TSH, mU/L	1.8 (1.0; 3.2)	1.9 (1.2; 3.0)	0.931^†^
Total PSA, ng/mL	21.8 (10.5; 34.6)	2.4 (1.1; 5.4)	<0.001^†^
Free PSA, ng/mL	1.8 (0.5; 4.3)	0.4 (0.2; 0.9)	0.001^†^
Free/total PSA ratio	8.0 (5.4; 16.1)	19.7 (13.0; 26.1)	<0.001^†^

BMI: body mass index; BPH: benign prostatic hyperplasia; GT: gamma-glutamyl transferase; HbA1c: glycated hemoglobin type-A1c; HDL: high-density lipoprotein; HOMA: homeostasis model assessment; LDL: low-density lipoprotein; PCa: prostate cancer; PSA: prostate-specific antigen; SGOT: serum glutamic-oxaloacetic transaminase; SGPT: serum glutamic-pyruvic transaminase; TC: total cholesterol; TGL: triglycerides; TSH: thyroid stimulating hormone; VLDL: very low-density lipoprotein. Data are expressed within each group as mean ± standard deviation or median with interquartile intervals in brackets. *P*^*∗*^ values for comparison of differences are calculated using Student's *t*-test, exception is for the use of the Mann–Whitney test^†^ for nonparametric data.

**Table 2:** Correlation of levels and of genotypic groups for MMP-1 with clinical traits at admission.

	Age	BMI	HbA1c	TGL	TC
	(years)	(kg/m^2^)	(%)	(mg/dL)	(mg/dL)

rs495366, GG vs AG vs AA	−0.03; 0.709	0.02; 0.813	−0.00; 0.945	−0.05; 0.474	0.00; 0.957
rs495366, GG vs A_	−0.01; 0.884	0.00; 0.960	−0.02; 0.768	−0.02; 0.745	0.02; 0.766
rs495366, G_ vs AA	−0.07; 0.332	0.06; 0.457	0.06; 0.436	−0.12; 0.094	0.06; 0.396
MMP-1, pg/mL	0.05; 0.511	0.05; 0.499	0.10; 0.181	0.05; 0.537	−0.03; 0.727

	Creatinin,	CRP,	TSH,	Total PSA,	MMP−1,
	mg/dL	mg/L	mU/L	ng/mL	ng/mL

rs495366, GG vs AG vs AA	−0.09; 0.237	−0.09; 0.249	0.03; 0.714	−0.06; 0.431	−0.14; 0.065
rs495366, GG *vs* A_	−0.06; 0.433	−0.06; 0.414	0.01; 0.935	−0.04; 0.605	−0.11; 0.134
rs495366, G_ vs AA	−0.14; 0.059	−0.13; 0.085	0.08; 0.252	−0.09; 0.208	−0.15; 0.045
MMP-1, ng/mL	0.11; 0.154	0.10; 0.168	0.01; 0.883	0.02; 0.753	−

Spearman's correlation test was used. Data are expressed in correlation index and significance level (two digits represent *r*; three digits represent *P*). BMI: body mass index; HbA1c: glycated hemoglobin type-A1c; MMP-1: matrix metalloproteinase-1; TGL: triglycerides; TC: total cholesterol; TSH: thyroid stimulating hormone; PSA: prostate-specific antigen. Individuals with the GG, AG, and AA genotypes were, respectively, represented by 1, 2, and 3 in the analysis with all groups, whereas G homozygotes and carriers of the A-allele were represented by 1 and 2 in this order in the subsequent analysis. Significance threshold was set at *P* ≤ 0.05.

## Data Availability

Data will be made available upon reasonable request.
